# Congenital Myasthenic Syndrome in Doberman Pinscher Dogs Is Associated with a Homozygous Missense Variant in *AGRN*

**DOI:** 10.3390/biom16081099

**Published:** 2026-07-28

**Authors:** G. Diane Shelton, Joan R. Coates, Janet E. Steiss, Ling T. Guo, Simon R. Platt, Katie M. Minor, Steven G. Friedenberg, Jonah N. Cullen, Garrett Bullock, Elizabeth A. Hansen, Martin L. Katz, Gary S. Johnson

**Affiliations:** 1 Department of Pathology, School of Medicine, University of California San Diego, La Jolla, CA 92093-0709, USA; liguo@health.ucsd.edu; 2 Department of Veterinary Medicine and Surgery, College of Veterinary Medicine, University of Missouri, Columbia, MO 65211, USA; coatesj@missouri.edu; 3 Scott-Richey Research Center, College of Veterinary Medicine, Auburn University, Auburn, AL 36849, USA; 4 Department of Small Animal Medicine and Surgery, College of Veterinary Medicine, University of Georgia, Athens, GA 30602, USA; srplatt1@gmail.com; 5 Department of Veterinary Clinical Sciences, College of Veterinary Medicine, University of Minnesota, St. Paul, MN 55108, USA; minork@umn.edu (K.M.M.); fried255@umn.edu (S.G.F.); cull0084@umn.edu (J.N.C.); 6 Canine Genetics Laboratory, Department of Pathobiology and Integrative Biomedical Sciences, College of Veterinary Medicine, University of Missouri, Columbia, MO 65211, USA; gebkd2@missouri.edu (G.B.); hansenl@missouri.edu (E.A.H.); katzm@health.missouri.edu (M.L.K.);

**Keywords:** canine, muscle, neuromuscular junction, post-synaptic membrane

## Abstract

Hereditary neuromuscular disorders in dogs can be difficult to classify since variants in different genes can result in similar clinical signs or variable phenotypes can be associated with the same DNA sequence variant. A disorder known for many years as Dancing Doberman Disease, suspected to be neuropathy or neuromyopathy, is characterized by repeated lifting and shifting of the pelvic limbs while standing and frequent sitting. More recently, Doberman Pinschers have been identified with a different and more severe phenotype characterized by a crouched stance and bunny hopping gait in the pelvic limbs that is termed duck walking. Dogs with both phenotypes show pelvic limb weakness, muscle atrophy, and fatigue, and clinical signs can progress to involve the thoracic limbs. These distinct phenotypes were evaluated clinically, histologically, and by whole-genome sequencing and genotyping a large cohort of affected and unaffected Doberman Pinschers. The same homozygous missense variant in *AGRN* (Dog 10K Boxer Tasha chr5:56,346,611,G>A; p.R1710H, XP 038377340.1) was associated with both disorders. *AGRN* encodes Agrin, an essential synaptic protein, that mediates clustering of acetylcholine receptors on the post-synaptic membrane at the neuromuscular junction. Variants in *AGRN* are associated with a congenital myasthenic syndrome (CMS) in humans. This is the first report of a CMS in dogs associated with an *AGRN* variant and expands the spectrum of known CMS genetic risk factors in this species. This study also highlights the importance of whole-genome sequencing (WGS) to accurately classify neuromuscular diseases as forms of CMS, which is not possible based on clinical presentation alone.

## 1. Introduction

Congenital myasthenic syndromes (CMSs) in people are a group of clinically and genetically heterogenous inherited disorders currently associated with variants in at least 40 different genes encoding neuromuscular junction (NMJ) proteins and compromising neuromuscular signal transmission [1]. CMSs are pathologically and functionally described as localizing to the synaptic, presynaptic, and post-synaptic regions of the NMJ. Causative genes are classified into 14 subtypes based on features such as endplate acetylcholine receptor (AChR) deficiency, defective AChR clustering, defective recycling of acetylcholine, glycosylation-deficient CMS, sodium channel CMS, and others [1]. Clinical signs of CMS are characterized by muscle weakness and fatigue with most CMS patients first exhibiting signs prior to 2 years of age, although the onset of CMS can occur at any age, including adolescence and adulthood [2]. Serum creatine kinase (CK) activities are usually normal or only mildly elevated, and AChR antibody titers are not elevated. Muscle biopsies may be normal or show variable changes including tubular aggregates, rimmed vacuoles, or central nuclei depending on the disease subtype. Repetitive nerve stimulation or single-fiber electromyography are utilized to diagnose CMS [2]. With the advent of next-generation sequencing, the spectrum of genetic risk variants associated with CMS has greatly expanded, and new disease syndromes continue to be identified [3,4].

CMS and the causative DNA sequence variants have been identified in several canine breeds. These include a missense variant in *ChAT*, the gene encoding choline acetyltransferase, in Old Danish Pointing Dogs (Gammel Dansk Hønsehund) [5]; in *CHRNE*, the gene encoding the epsilon subunit of the AChR in Jack Russell Terriers [6], Smooth Fox Terriers and English Springer Spaniels [7], and Heideterriers [8]; and in the gene encoding the collagenous tail of acetylcholinesterase (*COLQ*) in Labrador Retrievers [9] and Golden Retrievers [10]. In all three variants in all six breeds, age of onset was approximately at 6–12 weeks, and the disorders were characterized by persistent and generalized weakness or episodic weakness.

Over the past years, Doberman Pinschers exhibiting two distinct clinical phenotypes were observed: one in which affected adult dogs alternately lifted the pelvic limbs off the ground while standing suggesting the name “Dancing Doberman Disease”(DDD) [11,12], and a second clinical phenotype showing affected adult dogs walking with the pelvic limbs crouched suggesting the name “Duck Walking Dobermans” (DWD). Generalized weakness was not evident, but distal muscle weakness and abnormal fatigue were noted in both disorders. A non-specific myopathy, neuropathy, or neuromyopathy was suspected to underline each of these presentations. Studies were undertaken to identify the molecular basis of these disorders.

## 2. Materials and Methods

### 2.1. Animals

Doberman Pinschers that were evaluated in this study for clinical signs of DDD or DWD were client owned and examined by board certified veterinary neurologists or general veterinary practitioners in clinical veterinary practices. Oral and written consent were obtained from dog owners prior to pursuing diagnostic testing. Six Doberman Pinschers (Cases 1, 2, and 8–11) of both sexes and ranging in age from 2 to 8 years with clinical signs of DDD and five male Doberman Pinschers (Cases 3–7) ranging from 11 months to 6 years of age and with clinical signs of DWD were evaluated (Table 1).

### 2.2. Neurological Examination

The neurologic examination consisted of observation of mentation, posture and gait, cranial nerve evaluation, postural reaction testing, spinal reflexes, spinal palpation, and pain assessment. Seven affected Doberman Pinschers (Dogs 1–7) were anesthetized for electrodiagnostic testing.

### 2.3. Electrodiagnostic Testing

Electromyography (EMG), measurement of motor nerve conduction velocity (MNCV), and repetitive nerve stimulation (RNS) were performed as previously described [13,14]. A 26-gauge concentric needle electrode (Cadwell, Kennewick, WA; MedEnvoy Switzerland) was used for EMG recording. Major muscle groups in the thoracic and pelvic limbs as well as epaxial and head regions were examined at 2–4 depths. The muscle was considered abnormal if spontaneous activity was recorded on repeated attempts.

The compound muscle action potential (CMAP, M wave) was recorded from the extensor digitorum brevis (EDB) or interosseous muscle after stimulation of the sciatic–fibular or tibial nerve at the hip, stifle, and hock [13,15]. Here, 26-gauge monopolar needle stimulating electrodes (Ambu Neuroline A/S, Malaysia) were positioned caudally and deep to the greater trochanter of the femur, caudal to the fibular head or popliteal fossa, and caudally and deep to the tendon of the extensor digitorum longus or caudal to the gastrocnemius tendon to stimulate the proximal sciatic, fibular or tibial, and deep peroneal or distal tibial nerves. Cathodal stimulating electrodes were positioned one centimeter distal to anodal electrodes. A concentric needle recording electrode was inserted into the EDB or interosseous muscle, and the ground electrode was placed subdermal over the tuber calcaneus. Motor nerve conduction velocity (NCV) was calculated by dividing cathodal electrode distance (millimeters) by the difference in proximal and distal latency (milliseconds). Electrical stimulation was applied as a pulse width 100 ms duration at a frequency of 1 Hz with an intensity 20% greater than that required to produce a CMAP of maximal amplitude. The CMAP amplitude was recorded from the largest negative peak to the largest positive trough value. Repetitive nerve stimulation was performed on the deep fibular or tibial nerve at the hock, and CMAP was recorded with a concentric needle electrode in the EDB or interosseous muscle. Repetitive stimulation of the nerve was performed at 3, 5, and 10 Hz for a train of 10 impulses with a display to permit measurement of the peak-to-peak (O-P) amplitude and duration of the phase. A decrementing response is a reproducible decline in the amplitude and/or area of the M wave or CMAP of successive responses to RNS. The percent decrement was recorded between the first and fifth wave. An abnormal response is a decrement of 10% or greater [16]. In a normal response, the configuration of the successive CMAPs would remain unchanged.

### 2.4. Histopathology and Histochemistry

Muscle +/− peripheral nerve specimens were collected under general inhalational anesthesia from two affected Dancing Doberman and five affected Duck Walking Doberman dogs. Muscle specimens were collected from limb muscles including the tibialis cranialis, gastrocnemius, triceps brachii, biceps femoris, and vastus lateralis. Specimens were chilled and then shipped under refrigeration to the Comparative Neuromuscular Laboratory, University of California, San Diego by an express service. Upon receipt, muscles were flash frozen in isopentane precooled in liquid nitrogen, cut into 8 µm sections, and then evaluated using a standard panel of histochemical stains and reactions as previously described [17]. Fixed fascicular biopsies from the common fibular nerve were also collected under the same anesthesia, immersion-fixed into 10% neutral buffered formalin, and shipped to the laboratory. Upon receipt, fixed nerves were transferred to 2.5% glutaraldehyde, post-fixed in 2% aqueous osmium tetroxide, and processed to araldite resin blocks. Semi-thin sections (1 µm) were cut and stained with toluidine blue and paraphenylenediamine (PPD) prior to light microscopic examination.

### 2.5. Whole-Genome Sequencing (WGS)

Genomic DNA from Doberman Pinschers with the DDD phenotype (Cases 8–11) was prepared from EDTA-anticoagulated blood as previously described [18] and submitted to the University of Missouri Genomics Technology Core facility for library preparation and 150 bp paired-end sequencing on an Illumina NovaSeq 6000 sequencer, (Illumina, San Diego, CA, USA). Sequence reads were aligned against the Dog10K_Boxer_Tasha (GCA_000002285.4, Ensembl Release 112) reference assembly [19], with a mean coverage depth of 33.65. A previously described processing pipeline was used for variant calling and analysis jointly with sequence reads from 334 additional canine whole-genome sequences previously generated by the University of Missouri Canine Genetics Laboratory that were used as unaffected controls [20]. Whole-genome sequences have been deposited to the NCBI Sequence Read Archive and are available under BioProject accession PRJNA263947. BioSample IDs for the affected Doberman samples are SAMN10940812, SAMN12700542, SAMN13655919, and SAMN13655923.

Genomic DNA from Case 3 that exhibited the DWD phenotype was prepared from archived frozen biceps femoris muscle using the DNEasy kit (Qiagen, Germantown, MD, USA) according to supplier instructions. DNA libraries were prepared using an Illumina TruSeq PCR-Free kit, and 150 bp paired-end reads were generated on an Illumina HiSeq X sequencer by Azenta Life Sciences (South Plainfield, NJ, USA). A total of 440 million paired-end reads were generated, corresponding to a mean 26-fold genome-wide coverage available on NCBI’s Sequence Read Archive at SRR35635716. Sequence reads were mapped against the dog reference genome CanFam3.1 [21] and processed using allele frequency analysis on Case 3 compared to six presumed unaffected Dobermans, one “risk” neurologic Doberman (D02509), and 515 control non-Doberman dog genomes (Supplemental Table S1). Raw sequence reads are available under NCBI BioProject PRJNA937381. 

### 2.6. Genotyping

EDTA whole blood from 18 Doberman Pinschers of the DDD and 10 of the DWD phenotypes, 28 unaffected Dobermans, and 1047 Dobermans of unknown phenotypes serving as population controls, and 536 dogs of other breeds were submitted to the University of Missouri Canine Genetics Laboratory for genotyping (Table 2, Supplemental Table S2). DDD and DWD phenotype classification was based on a review by a veterinary neurologist (JRC) of clinical signs reported by the dogs’ owners. A custom TaqMan SNP Genotyping Assay (Applied Biosystems, Waltham, MA, USA) was designed to distinguish the G (reference) and A variants at *AGRN* (chr5:56.346.611, Dog10K_Boxer_Tasha reference coordinate). For this assay, PCR primer sequences were 5′-TGTCACTGGCGTTGCATGA-3′ and 5′-AGGGCGCACCTGATGAC-3′, and the competing probe sequences were 5′-VIC-CTTGGAATTCCGCTATGAC-NFQ-3′ (reference allele) and 5′-FAM-CTTGGAATTCCACTATGAC-NFQ-3′ (variant allele). Assays were performed in 25 µL reaction mixtures consisting of the DNA sample, 72 µM primers, 16 µM probes, and TaqMan Universal PCR Master Mix (Thermo Fisher Scientific, Waltham, MA, USA) on a StepOnePlus Real-Time PCR System (Applied Biosystems).

## 3. Results

### 3.1. Animals—History and Physical and Neurological Examinations

Signalment and phenotypes of 11 Dobermans with a clinical diagnosis of DDD or DWD that underwent electrodiagnostic, tissue biopsy examinations, or WGS are shown in Table 1.

*Dancing Dobermans*: Case 1 is a 2-year-old female spayed Doberman Pinscher that presented to the University of Georgia Veterinary Teaching Hospital with a 3-month history of acute onset of non-progressive shifting pelvic limb lameness (Supplemental Video S1). Alternating flexion and extension while standing was followed by frequent sitting. The dog was still active and able to run. Case 2 is a 2-year- and 7-month-old male Doberman Pinscher that presented to a neurology referral practice for an acute onset of pelvic limb weakness and incoordination. Posture showed intermittent lifting of the left pelvic limb progressing to alternating flexion and extension of both pelvic limbs while standing and frequent sitting. At times, the toes were knuckled over in both pelvic limbs (Supplemental Video S2). Neurological examinations revealed normal postural reactions, spinal reflexes, and cranial nerves for Case 1 and postural reaction deficits and general proprioceptive ataxia for Case 2. Muscle atrophy was evident in the pelvic limbs of both dogs. No other abnormalities were identified on physical and neurological examination. A presumptive diagnosis of Dancing Doberman Disease was made for Case 1. A spinal MRI from T3 to the sacrum was performed on Case 2 with no abnormal findings. An AChR antibody test on Case 2 was negative. A presumptive diagnosis in Case 2 was an L4-S3 myelopathy.

Cases 8–11 with DDD phenotypes similar to those of Cases 1 and 2 were used for whole-genome sequencing. The onset of signs was unknown in three dogs and 8 years in one dog. Three dogs were male neutered, and one dog was female spayed. Ages at sample submission ranged from 5 to 7 years and unknown in one dog.

*Duck Walking Dobermans*: Case 3 is a 2-year-old male neutered Doberman Pinscher that presented to Texas A&M University for an acute onset (within 24 h) of an abnormal pelvic limb gait and posture with persistent flexion in the stifles and tarsi and a waddling crouched gait (Supplemental Video S3). Spinal reflexes and postural reactions were normal in all limbs. A myelogram at the L5-6 subarachnoid space and two MRI examinations of the thoracic and lumbar spine were performed with no abnormalities identified in the spinal cord, nerve roots, or spinal nerves. Case 4 is a 6-year-old male Doberman Pinscher that presented to the University of Georgia Veterinary Teaching Hospital for evaluation of a 6-month history of crouched gait and stance. Just prior to referral, the dog had a T13-L2 laminectomy with no change in the pelvic limb stance after surgery. Spinal reflexes were absent in the pelvic limbs. Postural reactions were intact when weight was supported. The tentative diagnosis was a neuromuscular disorder. Case 5 is a 4-year- and 8-month-old male neutered Doberman Pinscher that presented to the University of Missouri Veterinary Health Center for a 2-year duration of progressive pelvic limb weakness, thoracolumbar kyphosis, a chronic history of an abnormal crouched gait and posture, and a preference to sit. Postural reaction deficits were not detected on neurological examinations. MRI of the thoracolumbar spine was normal. The AChR antibody test was negative. The tentative diagnosis was a neuromuscular disease. Cases 6 and 7 presented to private veterinary specialty practices at 4.5 years of age and 11 months of age, respectively, with several month histories of an abnormal crouched gait and videos supporting the abnormal posture and gait.

### 3.2. Electrodiagnostic Testing

Dogs 1–7 were placed under general inhalational anesthesia for electrodiagnostic testing. Electrodiagnostic testing performed on DDD Case 1 showed no abnormal spontaneous activity by needle EMG, MNCV was slow at 43 m/s measured in the tibial nerve with normal amplitude of the CMAP, and normal RNS without an obvious CMAP decrement. Only EMG was performed on all major muscle groups of Case 2, showing abnormal spontaneous activity including fibrillation potentials in the pelvic limb interosseous muscles. 

**Figure 1:**
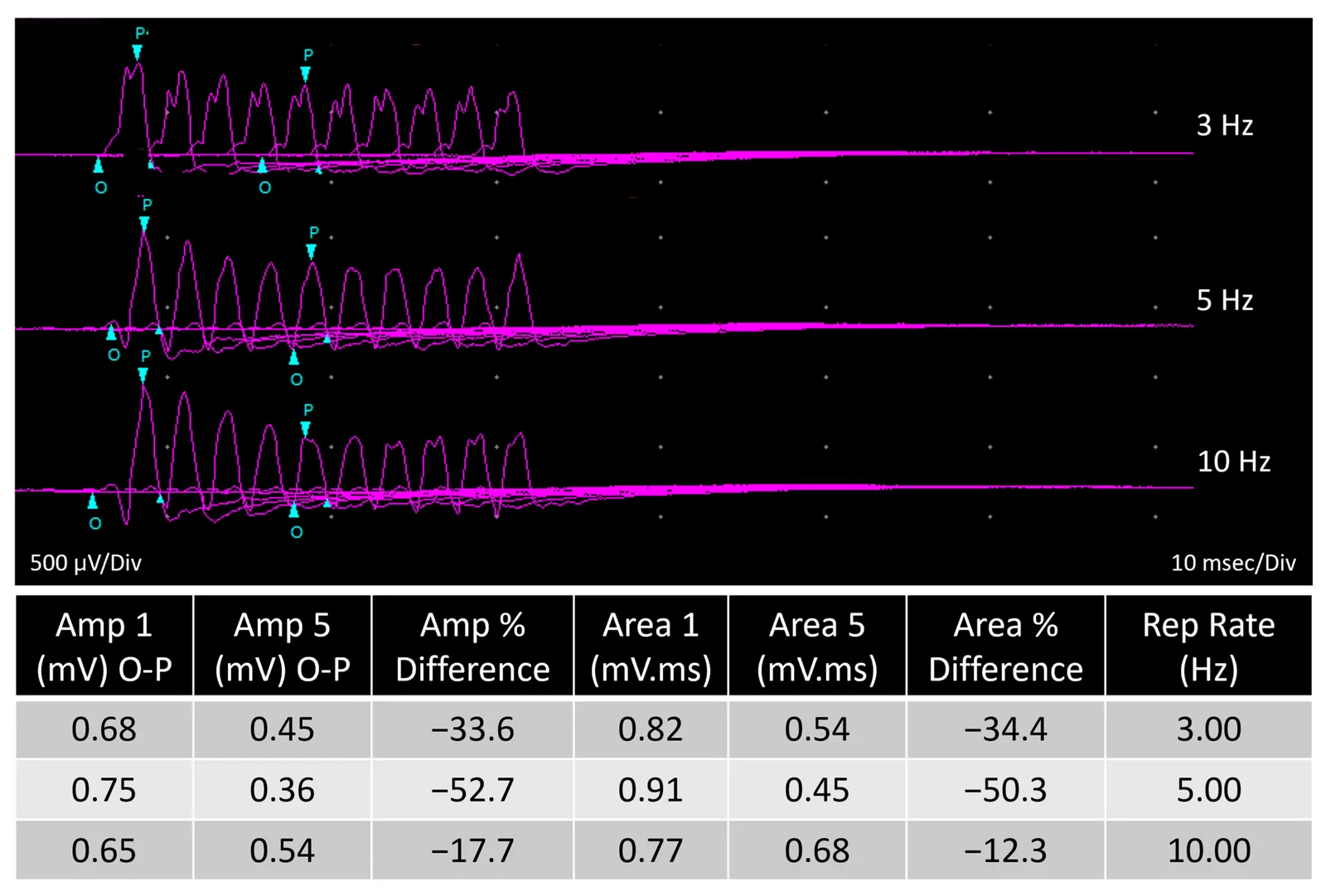
Repetitive nerve stimulation (RNS) on Case 5, a 4-year- and 8-month-old male neutered Doberman Pinscher. Successive compound muscle action potentials (CMAP, M wave) were recorded with a concentric needle electrode in the extensor digitorum brevis muscle during repetitive stimulation of the deep fibular nerve at a rate of 3, 5, and 10 Hz with a display to permit measurement of the peak-to-peak (O-P) amplitude and duration of the phase. Results show >10–20% decrementing response between the CMAP amplitude/area 1 and 5 after 10 supramaximal stimulations at 3, 5, and 10 Hz. In a normal dog, the configuration of the successive CMAPs would remain unchanged. O, onset latency; P, peak latency.

Electrodiagnostic testing was performed on DWD Cases 3–6. Case 3 showed diffuse EMG changes in all muscles tested with 1+ fibrillations and positive sharp waves. Motor NCV was slow at 48 m/s in both the fibular and ulnar nerves. Repetitive nerve stimulation did not show a decremental response. Case 4 showed normal motor NCV with average conductance across the tibial nerve at 70 m/s. RNS was not performed. For Case 5, electromyography was performed on the left side of major muscle groups in the limbs, epaxial, and head and showed no spontaneous activity. A nerve conduction study of the sciatic- fibular nerve showed mild slowing of the conduction velocity distal to the stifle. Conduction velocities from hip to stifle and stifle to hock were 69 m/s (reference range, 76 +/− 10 m/s) and 44 m/s (reference range, 68 +/− 11 m/s), respectively. CMAP amplitudes after stimulation were decreased at the hip (4.3 mV), stifle (4.6 mV), and hock (3.7 mV). Repetitive nerve stimulation at the hock showed >20% decrement between waveforms 1 and 5 after 10 stimulations at 5 Hz (Figure 1). Only EMG was performed on Cases 6 and 7 with patchy and marked fibrillation potentials and positive sharp waves in some muscle groups.

### 3.3. Histopathology and Histochemistry

**Figure 2:**
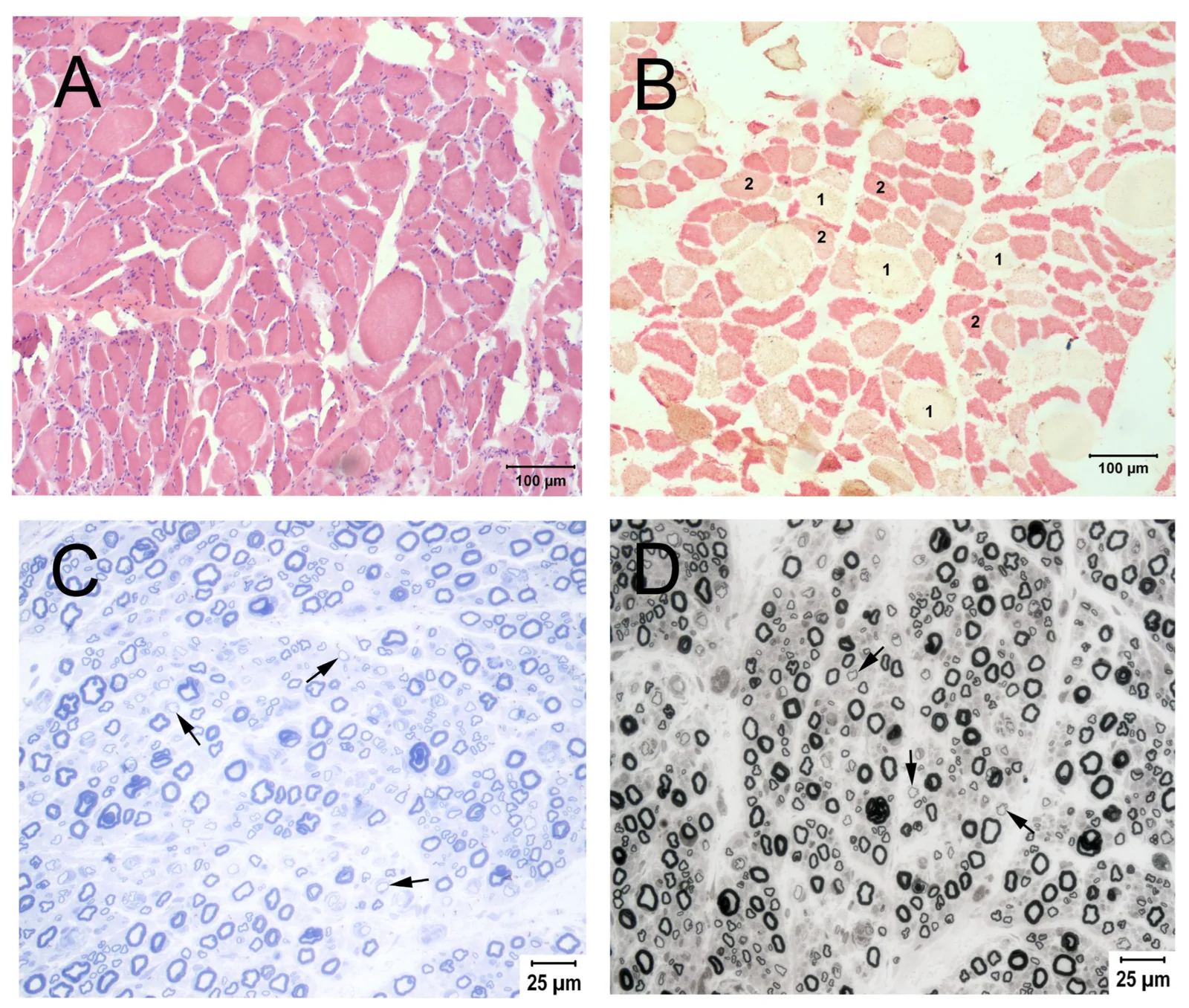
Cryosections (8 µm, (**A**,**B**)) from the gastrocnemius muscle of DDD Case 1 showing moderate generalized myofiber atrophy ((**A**), H&E stain). Atrophic fibers were of both fiber types ((**B**), immunostaining for type 1 [light tan stain] and type 2 [pink-stained fibers]). Atrophic fibers had an anguloid to angular shape consistent with denervation. Resin embedded sections from the common fibular nerve (1 μm, (**C**,**D**)) showed large nerve fiber loss and numerous small caliber, thinly myelinated fibers ((**C**), is stained with toluidine blue and (**D**) is stained with PPD for myelin). Arrows in (**C**,**D**) highlight small caliber, thinly myelinated fibers. Neuropathy secondary to large nerve fiber loss was suspected based on muscle and nerve biopsies.

For DDD Case 1, biopsies from the gastrocnemius (Figure 2A,B) and biceps femoris muscles showed moderate generalized myofiber atrophy (Figure 2A). Atrophic fibers were of both histochemical type 1 and type 2 in a pattern consistent with denervation (Figure 2B). Moderate large nerve fiber loss and numerous small thinly myelinated fibers were present in the common peroneal nerve (Figure 2C,D). Neuropathy resulting from large nerve fiber loss was suspected. Biopsies from the tibialis cranialis and rectus femoris muscles were submitted from DDD Case 2 and showed only scattered atrophic fibers in a pattern suggestive of mild denervation. A nerve biopsy was not submitted.

**Figure 3:**
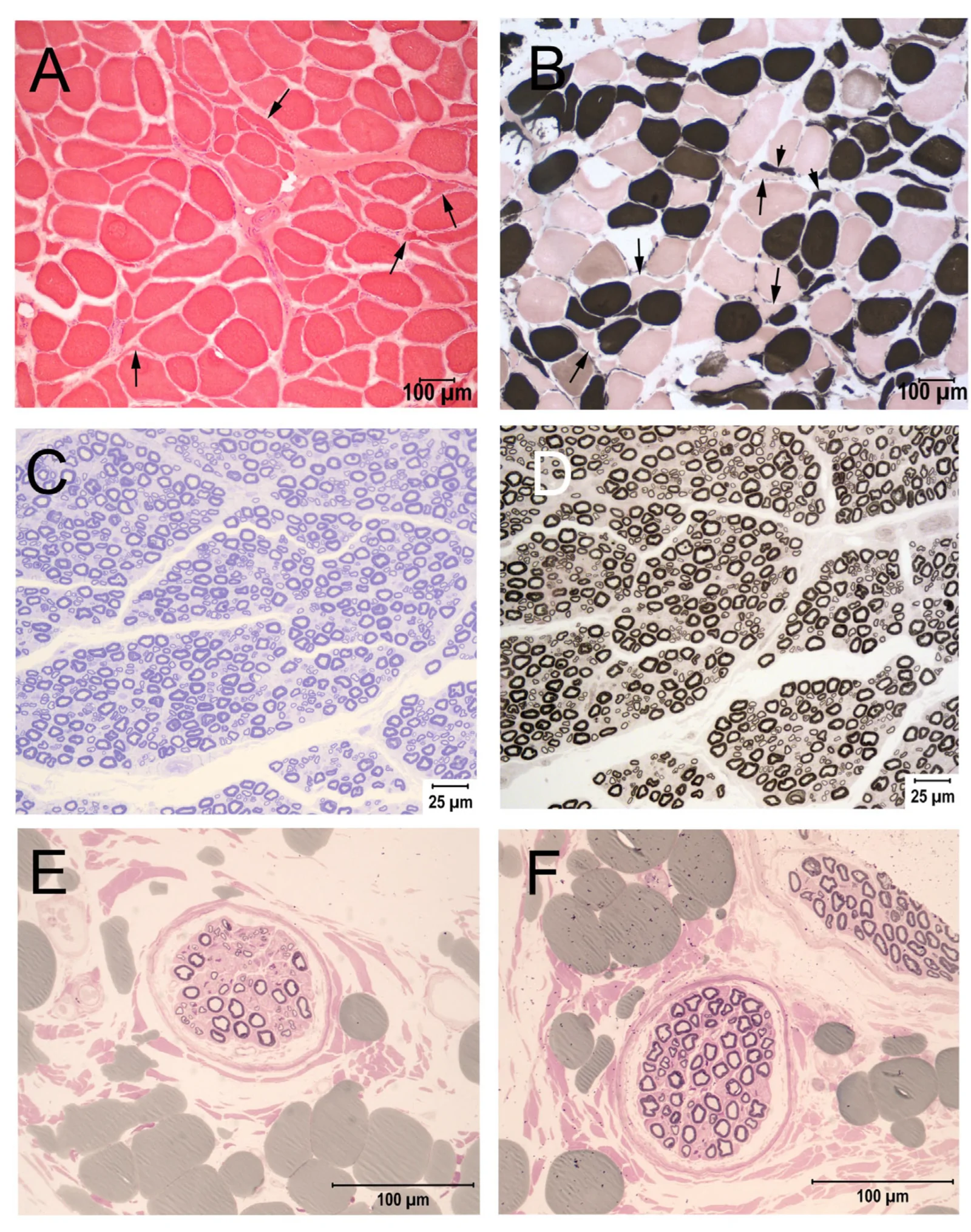
Cryosections from the biceps femoris muscle of DWD Case 5 showing scattered and small groups of atrophic fibers having an angular shape (arrows in (**A**), H&E stain) and of both fiber types (arrows in (**B**), short tailed arrows highlight darkly stained type 1 fibers and long tailed arrows show light staining type 2 fibers; myofibrillar ATPase reaction at pH 4.3 where type 1 fibers are dark and type 2 fibers are light). The pattern of angular atrophied fibers of both fiber types is consistent with mild or early denervation. In images (**C**,**D**), resin sections (1 μm) from the common fibular nerve of Case 5 are stained with toluidine blue (**C**) and ppd (**D**). Nerve fiber density is subjectively appropriate in both images. In Case 6 (**E**,**F**), large nerve fiber loss was variable in medium sized fascicles with loss of large, myelinated fibers in some fascicles (**E**) and normal myelinated fiber density in other medium size fascicles (**F**). Subperineurial edema was prominent in the medium size fascicles (**E**,**F**).

For DWD Case 3, a pattern of muscle fiber atrophy consistent with mild denervation was present in the tibialis cranialis, vastus lateralis, and triceps brachii muscles, with no abnormalities identified in the common fibular nerve. Similar findings were present in the biceps femoris muscle from Case 4 and the biceps femoris and gastrocnemius muscles of Case 5 (Figure 3), with no abnormalities (Case 4) or only mild large fiber loss (Case 5) identified in the common fibular nerve. Muscle biopsies from the triceps brachii and tibialis cranialis muscles of Case 6 similarly showed variability in myofiber size with atrophic fibers having an angular shape consistent with denervation. Variable large nerve fiber loss was identified in the medium size fascicles from the common fibular nerve (Figure 3). Except for variability in myofiber size, no specific abnormalities were identified in the biopsy from the vastus lateralis muscle of Case 7. A peripheral nerve biopsy was not submitted.

In both the DDD and DWD affected dogs, muscle biopsies showed only mild and non-specific changes such as variability in myofiber size or a pattern of muscle fiber atrophy consistent with mild denervation. The common fibular nerve was typically biopsied, and large nerve fiber loss was observed.

### 3.4. Genetic Testing

**Figure 4:**
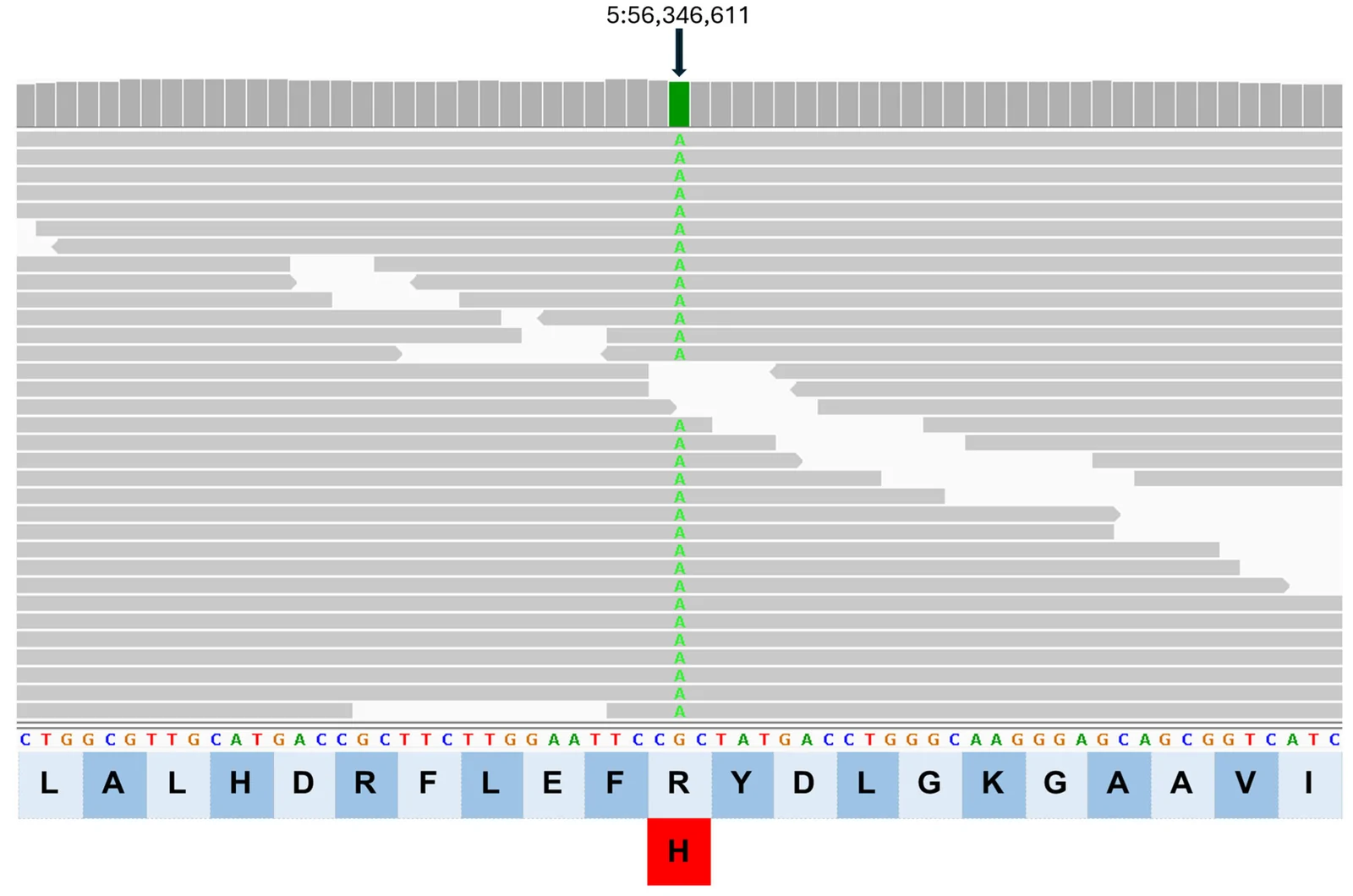
Integrative Genomics Viewer (IGV) image of sequence reads from a DDD dog flanking *AGRN* (chr5:56,346,611G>A; p.R1710H, XP_038377340.1; arrow). The reference amino acid sequence is shown below the nucleotide sequence. The G to A change in the nucleotide sequence predicts an R to H change in the protein amino acid sequence.

Whole-genome sequencing was performed on Cases 8–11. All four dogs had the DDD clinical phenotype. Called variants were first sorted by minor allele frequency (MAF) and then filtered for variants in the homozygous state in all four affected dogs. A unique homozygous missense variant in *AGRN* (Dog10K_Boxer_Tasha chr5:56,346,611G>A; p. R1710H, XP_038377340.1) was identified all four dogs. Variant calling was confirmed by visual inspection using the Integrative Genomics Viewer (IGV, Figure 4) [22]. The *AGRN* variant was not found in any of 754 additional canine whole-genome sequences previously generated from numerous dogs by the University of Missouri Canine Genetics Laboratory that served as unaffected controls. This included five Doberman Pinschers that did not exhibit either disease phenotype (Table 2).

Sequence reads generated by WGS from Case 3 with the DWD phenotype were mapped against the dog reference genome CanFam 3.1. A list of high (e.g., frame shift, loss or gain of stop or start codon, affecting a splice junction) and moderate impact (e.g., missense) variants identified in Case 3 were compared to a reference population of 522 other WGS generated at the University of Minnesota (Supplemental Table S3). A homozygous missense variant in *AGRN* (CanFam3.1 chr5:56,268,779 G>A; p.R1710H, XP 005620417.1) was identified. Variant calling was confirmed by visual inspection using the IGV (Supplemental Figure S1). Within the University of Minnesota reference population of 522 other dogs, there was one heterozygous Doberman Pinscher, as well as two homozygous Doberman Pinschers including a dog with vestibular signs beginning at 11 weeks of age and a dog with congestive heart failure due to dilated cardiomyopathy presenting at 8 years of age. Neither dog had clinical signs of the DDD or DWD phenotype. After accounting for differences in the reference assembly annotations used for the DDD and DWD dogs, the variant was identical for both DDD and DWD.

### 3.5. Genotyping of Doberman Pinscher Dogs

To assess concordance between the *AGRN* genotype and the DDD/DWD clinical phenotypes, records were reviewed on the 56 Dobermans that were genotyped for the *AGRN* variant with phenotypic information available, as well as a large cohort of 1047 Dobermans of undetermined phenotype (Table 2). Of 31 dogs that were homozygous for the risk allele, 28 exhibited signs of DDD or DWD. Of the three remaining dogs, one was 5 years of age at the time of evaluation, and the other two had health issues that may have masked DDD/DWD clinical signs. Of the 28 Dobermans clinically affected with the DDD/DWD phenotypes, all 28 were homozygous for the A allele. The remaining 25 unaffected dogs that were heterozygous or homozygous for the G allele did not show clinical signs of disease. Of 1047 Dobermans of undetermined phenotype, 34 (3.2%) were homozygous for the A allele, and 259 (24.7%) were heterozygous. A total of 536 dogs of 53 other breeds were also genotyped for the variant. All these dogs were homozygous for the reference allele (Supplemental Table S2).

The *AGRN* genotype was significantly associated with the disease phenotype (Chi-square = 45.2; *p* < 0.001). The risk allele frequency among the large cohort of population controls was 0.16. An allele frequency of 0.16 calculated from more than 1000 dogs archived with the CHIC DNA repository indicates that the variant is present at a significant level within the breed more broadly. In fact, the variant allele frequency among a large cohort of Dobermans was over 15% (Table 2), indicating that the risk allele is widespread in this breed.

## 4. Discussion

In this report we described a previously unknown *AGRN* variant in adult Doberman Pinschers that is significantly associated with two different forms of CMS. Compared to previously reported dogs with CMS [5,6,7,8,9,10], the clinical presentations were unusual. Two distinct phenotypes were noted. One phenotype was noted in affected adult dogs that alternately lifted the pelvic limbs off the ground while standing, suggesting the name “Dancing Doberman Disease” [11,12] (Supplemental Videos S1 and S2). Adult dogs with the second clinical phenotype walked with the pelvic limbs crouched, suggesting the name “Duck Walking Dobermans” [Supplemental Video S3]. Generalized weakness was not evident, but distal muscle weakness was apparent. It was not clear prior to identification of the *AGRN* variant that these were differing presentations of the same disease, and CMS was not even considered as a differential diagnosis. Previously described cases of canine CMS present with generalized weakness at very young ages [5,6,7,8,9,10]. Recent studies have demonstrated significant phenotypic heterogeneity in human CMS among subjects with the same causal DNA sequence variants [23]. In addition, two distinct phenotypes, hemiplegic migraine and episodic ataxia type 2, were caused by the same novel *CACNA1A* variant [24]. Unknown modifying genes and gene interactions, epigenetics, or environmental factors may play a role in clinical variability [25].

A homozygous missense variant in *AGRN* (Dog10K_Boxer chr5:56,346,611G>A; p. R1710H) was identified in all four DDD dogs with WGS performed at the University of Missouri (Cases 8–11) and in the DWD (Case 3) with WGS analyzed at the University of Minnesota. The missense variant identified in the *AGRN* gene was predicted to be deleterious via pathogenicity prediction programs E-SNPs&GO [26], Provean [27], and PolyPhen-2 [28]. The protein product of *AGRN*, Agrin, is a large molecule secreted from the nerve terminal at the NMJ and carries binding domains for laminins, neural cell adhesion molecule, α-dystroglycan, and LRP4 [2]. Pathogenic *AGRN* variants impair AChR clustering and anchoring at the NMJ. Most human AGRN-CMS patients develop muscle weakness beginning in childhood, and symptoms range from mild muscle weakness in the lower limbs to severe generalized muscle weakness that requires respiratory support [2]. Albuterol and ephedrine have been described as effective treatments in human AGRN-CMS patients [2]. Case 2 (DDD) and Case 5 (DWD) were treated with albuterol, and both had transient improvement in clinical signs.

All cases affected with DDD and DWD were homozygous for the AGRN variant; thus, this variant is most likely a major contributing factor to development of the disease. In addition to the variability in clinical signs, there was a wide range in the age of onset, suggesting that there are likely other genetic factors that modulate the disease presentation. The Dobermans that were homozygous for the at-risk allele that did not show clinical signs of either disease may have been too young for clinical signs to be evident since in some cases the signs did not become obvious before 7 to 8 years of age. Two of these dogs had other concurrent diseases that may have masked the expected clinical signs of DDD/DWD or may have had mild disease for which a full neurologic evaluation was not performed. None of the dogs that were heterozygous or homozygous for the reference G allele showed clinical signs of disease, indicating that the presence of one normal allele may be sufficient to prevent development of the disorder. Based on genotyping many Doberman Pinschers of unknown phenotype whose DNA was archived in the CHIC DNA Repository (https://ofa.org/chic-programs accessed on 25 July 2026), the risk allele appears to be fairly common in the breed (Table 2). In total, 34 of 1047 dogs were homozygous for the risk allele. Thus, it should be possible to identify a larger number of homozygous dogs and determine whether they are exhibiting signs to more completely assess the degree of phenotype penetrance for this variant. Disease penetrance is an estimate of how many homozygous dogs with the disease genotype show clinical signs of disease, typically by a certain age. Further, with development of a specific genetic test for the *AGRN* variant, WGS can be performed on a larger number of affected Dobermans with known phenotypes to search for potential genetic modifiers that can affect age of onset or clinical phenotype.

Pathogenic *AGRN* variants have been identified in Charcot–Marie–Tooth neuropathy type 2 (CMT2), a hereditary motor neuropathy [29]. In some of the Dobermans evaluated with electrodiagnostic testing, mild abnormal spontaneous activity was present in the muscles with mild to moderately slow motor nerve conduction velocities. Concurrent with these changes, a pattern of atrophy consistent with denervation was present in muscle biopsies and large nerve fiber loss in the peripheral nerves (Figure 1 and Figure 2) in both DDD and DWD phenotypes. Based on the known function of agrin, it is not apparent by what mechanism nerve fiber loss would occur in motor neurons. Agrin is secreted by the motor nerve terminal into the NMJ synaptic cleft where it mediates aggregation of AChRs on the adjacent muscle membrane. Impairment of this function would be expected to impede signal transmission from the motor neuron to the muscle. This in turn could reduce retrograde signaling from the muscle that is necessary to maintain motor nerve axon integrity. However, in mice with a missense variant in *Agrn* and a CMS phenotype, the axons of peripheral nerves did not retract or degenerate and did not exhibit any changes in morphology, axon number, or axon size [30]. Further research will be necessary to confirm the effect of the *AGRN* variant in the Dobermans on peripheral nerve axon integrity.

*AGRN* has at least four alternatively spliced mRNAs that generate different protein isoforms that are all affected by the variant identified in the DDD and DWD dogs (Supplemental Table S3) [31,32,33,34]. The isoforms differ in their tissue distribution and function. While one isoform expressed in motor neurons is primarily involved in clustering of acetylcholine receptors on the post-synaptic membrane at the NMJ, the other isoforms are expressed in different tissue and cell types, including muscle. An isoform expressed in muscle not directly involved in acetylcholine receptor clustering may be involved in maintaining muscle fiber integrity [35,36]. The effect of the G to A variant may affect this function and thereby contribute directly to the observed muscle pathology.

There are several limitations to this study. The clinical evaluations were performed by several different veterinary clinicians and specialists with varying diagnostic approaches. Protocols for electrodiagnostic testing were either varied or specific studies not performed on some dogs, including demonstration of a decremental response of the compound muscle action potential to repetitive nerve stimulation. The muscles and nerves sampled were not standardized. Following clinical evaluations, a CMS was not considered as a differential diagnosis, and appropriate muscle specimens were not collected for evaluation of the motor end plate. Despite these limitations, adequate clinical evaluations were performed to identify the two distinct phenotypes with WGS and bioinformatics on enough cases to show that both phenotypes were associated with the same *AGRN* variant. Now that genotyping can be performed to identify pre-symptomatic at-risk dogs and affected dogs, a more systematic study of disease-related nerve and muscle pathology and functional validation of the variant can be conducted.

## 5. Conclusions

This study demonstrates an expanded spectrum of clinical phenotypes that can be associated with a specific disease gene (*AGRN*) and the complexity of the pathogenesis of neuromuscular diseases. Pathogenic *AGRN* variants impair AChR clustering and anchoring at the NMJ. Electrodiagnostic testing and evaluation of muscle and peripheral nerve biopsies are essential for correct classification of neuromuscular diseases in addition to WGS and bioinformatics for confirmation of risk factors or causal gene variants. Pathogenic *AGRN* variants impair AChR clustering and anchoring at the NMJ and may be affected by a specific disease genotype.

## Figures and Tables

**Table 1:** Signalment on 11 Doberman Pinschers Evaluated for Clinical Signs of Dancing Doberman Disease (DDD) or Duck Walking Doberman Disease (DWD).

Case #	Age at Presentation	Sex	Duration of Clinical Signs	Phenotype
1	2 yrs	FS	3 mos	DDD
2	2 yrs 7 mos	M	Acute onset	DDD
3	2 yrs	MN	Acute onset	DWD
4	6 yrs	M	6 mos	DWD
5	4 yrs 8 mos	MN	16 mos	DWD
6	4 yrs 6 mos	MN	chronic	DWD
7	11 mos	MN	5 mos	DWD
8	Unknown	MN	Unknown	DDD
9	5 yrs	MN	Unknown	DDD
10	7 yrs	FS	Unknown	DDD
11	8 yrs	MN	Acute onset	DDD

**Table 2:** Phenotype, Genotype in Affected Dobermans, Unaffected Controls and in a Large Unphenotyped Cohort of Population Controls.

		Genotype		Number of Dogs
**Phenotype**	GG	AG	AA	
DDD/DWD	0	0	28	28
Unaffected	21	4	3	28
Population Controls	754	259	34	1047

## Data Availability

All genome sequences are publicly available under NCBI BioProject accession PRJNA263947 and under NCBI BioProject PRJNA937381.

## Supplementary Materials

The following supporting information can be downloaded at: https://www.mdpi.com/article/10.3390/biom16081099/s1, Figure S1: IGV image from Case 3 DWD; Table S1: Sample IDs for allele frequency analysis performed at the University of Minnesota; Table S2: Non-Doberman dogs genotyped homozygous for the AGRN reference allele at the University of Missouri; Table S3: Case 3, moderate and high impact variant allele frequency analysis against the University of Minnesota’s WGS database of 522 other dogs. The four transcripts of alternatively spliced mRNA are highlighted in yellow; Video S1: Case 1, a 2-year-old female spayed Doberman with clinical signs of DDD; Video S2: Case 2, a 2-year- and 7-month-old male neutered Doberman with clinical signs of DDD and proprioceptive deficits (knuckling of the toes) in the pelvic limbs; Video S3: Case 5, a 2 year- and 8-month-old female spayed Doberman with an acute onset of DWD.

## Abbreviations

The following abbreviations are used in this manuscript:

CMS	Congenital myasthenic syndrome
AChR	Acetylcholine receptor
*CHRNE*	Gene encoding the epsilon subunit of the AChR
*COLQ*	Gene encoding the collagenous tail of acetylcholinesterase
EMG	Electromyography
NCV	Nerve conduction velocity
RNS	Repetitive nerve stimulation
PPD	Paraphenylenediamine
WGS	Whole-genome sequencing
