# Supplementary material for: Congenital Myasthenic Syndrome in Doberman Pinscher Dogs Is Associated with a Homozygous Missense Variant in AGRN
Source: Biomolecules. 2026 Jul 28;16(8):1099. doi: 10.3390/biom16081099 (PMC13511124; doi:10.3390/biom16081099)
Supplement: Supplementary file 1 [file biomolecules-16-01099-s001.zip › Supplementary Figure S1. AGRN IGV CanFam3 Case 3.pdf]

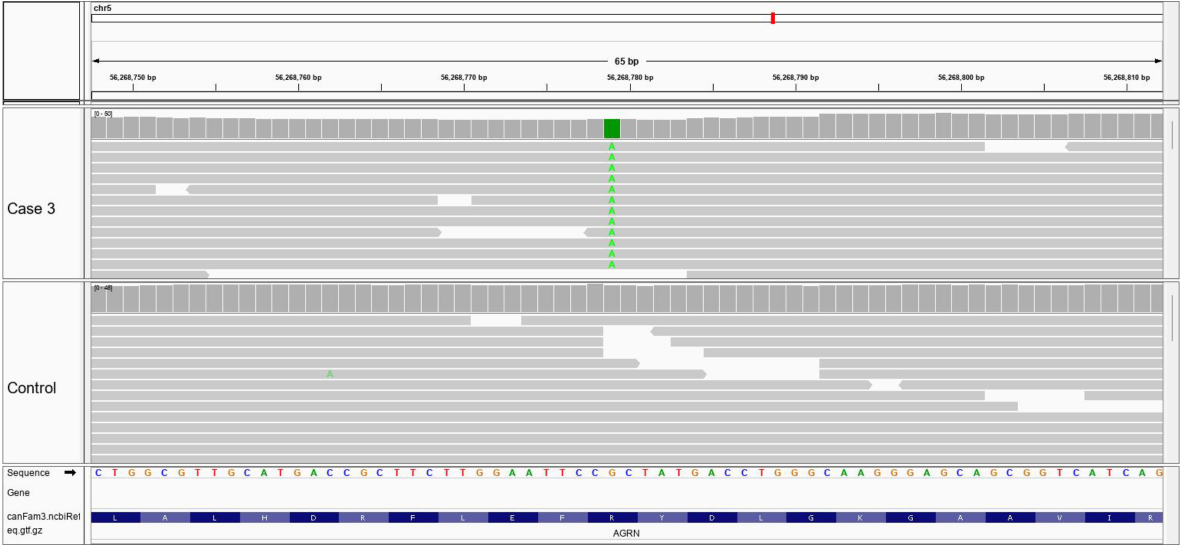

chr5
65 bp
56,268,750 bp
56,268,760 bp
56,268,770 bp
56,268,780 bp
56,268,790 bp
56,268,800 bp
56,268,810 bp
Case 3
[0 - 50]
Control
[0 - 48]
Sequence
C T G G C G T T G C A T G A C C G C T T C T T G G A A T T C C G C T A T G A C C T G G G C A A G G G A G C A G C G G T C A T C A G
Gene
canFam3.ncbiRefseq.gtf.gz
L A L H D R F L E F R Y D L G K G A A V I R
AGRN
